# Automatic localization of the prostatic urethra for image guided radiation therapy

**DOI:** 10.1016/j.tipsro.2021.05.002

**Published:** 2021-06-11

**Authors:** Nicola J. Nasser, Jonathan Klein, Eyal Fenig, Abed Agbarya

**Affiliations:** a Department of Radiation Oncology, University of Maryland School of Medicine, Maryland Proton Treatment Center, Baltimore, MD, USA; b Department of Radiation Oncology, Montefiore Medical Center and Albert Einstein College of Medicine, Bronx, NY, USA; c Institute of Oncology, Davidoff Center, Rabin Medical Center, Beilinson Hospital, Petah Tikva, Israel; d Institute of Oncology, Bnai Zion Medical Center, Haifa, Israel

## Abstract

Treatment of prostate cancer with radiation therapy (RT) requires image guided RT (IGRT) to focus the radiation on the target volumes while minimizing doses to organs at risk. Here we describe a urinary catheter that allows imaging of the prostatic urethra and uses it for automatic localization of the prostate for IGRT. The catheter has a contrast lumen that can be empty or full with contrast. Computerized tomography is performed twice, with contrast lumen empty and full, allowing urethral autosegmentation using digital subtraction. Under ultrasound, continuous urethral visualization is possible by pumping aerated gel in- and out of the contrast lumen.

## Highlights

• Prostatic urethra can be used for image guided radiation for prostate cancer.
• Computer “finds” the urethra by digital subtraction of scans with / without contrast.
• Urethra segmentation used to setup the patient and position prostate as in simulation.
• A catheter with continuous aerated gel flow is used to detect the urethra under US.

## Introduction

Prostate cancer is the most prevalent non-cutaneous malignancy among men [1]. External Beam radiation therapy (EBRT) for prostate cancer using conventional fractionation provides 1.8–2 Gy per fraction every day, 5 days a week, over 7–10 weeks [2]. Image guided radiation therapy (IGRT) is usually utilized for EBRT as the prostate location can change relative to the pelvic bones, as a function of rectal and bladder filling. Implantation of at least 3 fiducial markers in the prostate enables three dimensional localization of the prostate using daily 2D-kV or cone beam computerized tomography (CBCT) [2].

Prostate cancer has a low α/β ratio value that favors the use of hypofractionated radiotherapy schedules [3]. Multiple clinical trials have demonstrated that stereotactic body radiation therapy (SBRT) using doses of 7.5–9 Gy per fraction, for a total of five fractions, is safe and likely non-inferior to conventionally fractionated RT regimens [4], [5], [6], [7], [8], [9], [10], [11].

SBRT for prostate cancer is preferably performed with a full urinary bladder. When full, the bladder pushes part of the small intestine lying just above it superiorly and potentially away from the radiation therapy fields, reducing dose to the bowel, and also results in reduced dose to the walls of the bladder compared to the collapsed (empty) organ [12]. However, maintaining consistent bladder filling over multiple treatment fractions, to ensure accurate reproduction of patient positioning over the treatment course and to ensure adequate sparing of small bowel and bladder wall, can be challenging. We have recently published a description of an in-development catheter, the Nasser-Zelefsky catheter [12], that keeps the bladder full to a prespecified level and expels excess urine to maintain consistent bladder filling [12]. This catheter could be particularly useful during RT procedures that require relatively long delivery times, such as prostate SBRT or magnetic resonance imaging guided therapy [13]. In this report, we describe an additional novel device that allows automatic segmentation of the prostatic urethra using digital subtraction technology, and positions the patient automatically before EBRT to ensure that the prostate remains in the same location relative to the treatment isocenter as at the treatment planning computerized tomography (CT) scan [14]. This technology allows the computer to locate the prostatic urethra and use it for image guidance. Using the same mechanism, we describe a catheter for real-time identification of the prostatic urethra under ultrasound to facilitate its avoidance during brachytherapy and biopsy procedures.

## Nasser – Zelefsky CT catheter

The Nasser – Zelefsky CT Catheter (NZCC) is a new invention that uses digital subtraction technology to segment the prostatic urethra and to automatically move the treatment couch so that the prostate is in the same location relative to the treatment isocenter at each RT fraction. Digital subtraction technology is widely used in angiography to image blood vessels using fluoroscopy. In digital subtraction angiography, images of areas of interest are obtained before and after the introduction of contrast into the blood vessels. Images without contrast are then subtracted from the images with contrast, which results in an image of the blood vessels without the structures surrounding them [15], [16], [17], [18].

**Fig. 1:**
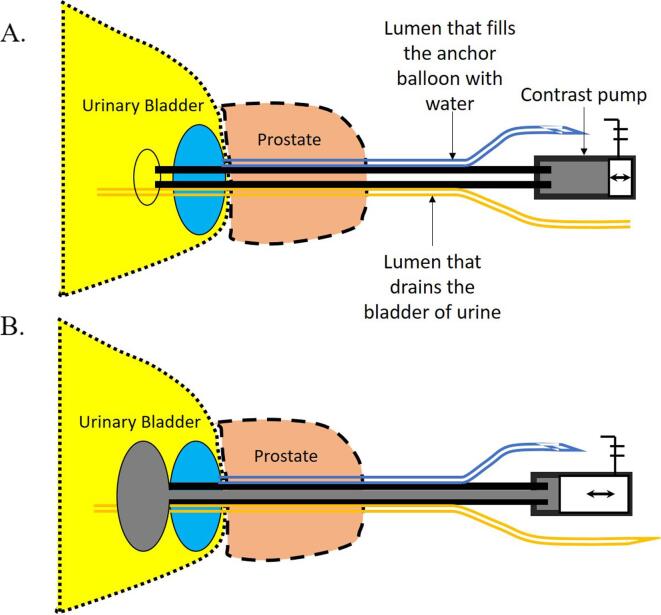
Schematic drawing of Nasser – Zelefsky CT catheter. The catheter has a balloon filled with water (Blue) to anchor the catheter to the bladder, and a contrast lumen that has its opening connected to a contrast pump. The contrast pump has an antenna that allows it to be remotely controlled. A. Contrast pump not activated; contrast lumen empty. B. Contrast pump activated; lumen full with contrast. (For interpretation of the references to colour in this figure legend, the reader is referred to the web version of this article.)

**Fig. 2:**
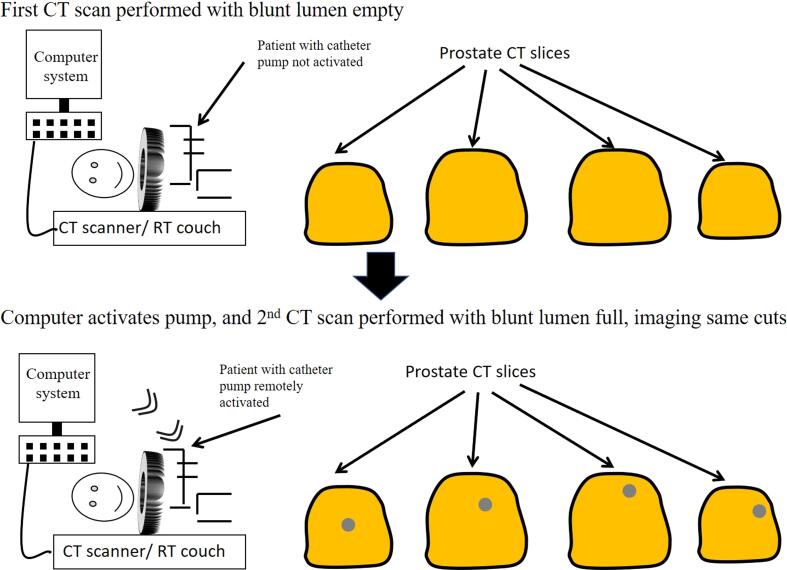
Nasser – Zelefsky CT catheter is inserted before CT simulation and each fraction of SBRT. First set of CT scans obtained before (upper panel) and after (lower panel) activation of the contrast pump. First and second scans are obtained within seconds of each other, capturing the same CT slices. Using digital subtraction, the urethra is segmented, and the patient is repositioned so that the urethra is in the same location relative to the isocenter at time of treatment, as at the time of CT simulation.

The NZCC employs the same technology to automatically position the patient for prostate IGRT. The catheter has three lumens [14] (Fig. 1). After insertion into the bladder, the first lumen fills a balloon with water to anchor the catheter inside the bladder. The second lumen drains the bladder of urine (Fig. 1). The third lumen has an external opening attached to a contrast pump (Fig. 1) controlled remotely by the computer system that controls the CT scanner, while its other end drains in a reservoir balloon that allow the insertion of contrast into the lumen while maintaining its low internal pressure (Fig. 1 and Fig. 2).

The NZCC is inserted by a medical provider before CT simulation or SBRT and can be inserted immediately before each fraction and removed afterward to reduce the risk of infection. After the first scan of the pelvis is obtained (Fig. 2A), the computer system activates the contrast pump attached to the contrast lumen, filling the third lumen with contrast (Fig. 2B). Immediately after, a repeat CT scan of the same area is obtained. This results in two sets of CT images (Fig. 2), one with the urethra empty and the second with the urethra full of contrast. The computer system then subtracts the first image set from the second set, resulting in automatic segmentation of the urethra, with a resolution that is a function of the slice thickness. This process is repeated at CT simulation and before each fraction of radiation.

Before each fraction of radiation, the pelvis is imaged with the catheter empty. The computer system then activates the filling of the catheter third lumen with contrast, and re-images the pelvis again using the same CT slices. Urethral segmentation is done by subtracting the first image set from the second. Once the pre-treatment segmentation has been performed, the computer system moves the couch so that the prostatic urethra location matches its position relative to the isocenter from the CT simulation scan. Although we have described the technique using CT guided therapy, it could be used for KV 2D guided imaging as well.

The NZCC could replace the need for fiducial markers implantation in the prostate. The number of fusion points employed in this technology is a function of CBCT slice thickness and prostate size, resulting in 5–50 points of reference for image matching, potentially increasing the fusion accuracy. Also, because the process is computerized, prostate localization and couch repositioning, are automatic, potentially resulting in fewer human errors and decreased daily setup time.

## Nasser – Zelefsky ultrasound catheter

**Fig. 3:**
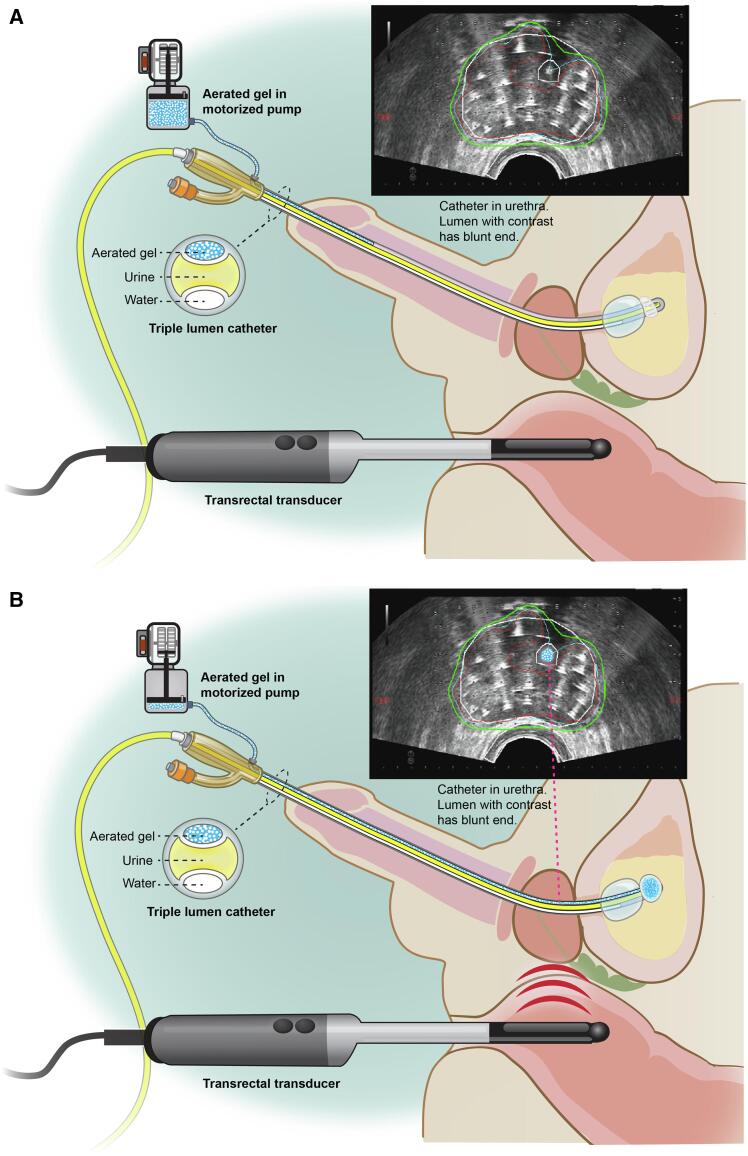
Nasser – Zelefsky US catheter. A triple lumen catheter. The first lumen fills a balloon with water that anchors the catheter to the bladder. Second lumen drains the bladder of urine. Third lumen connects to a pump that continuously drives aerated gel in and out of that lumen. The lumen ends in a small balloon designed to decrease the tension inside it when the aerated gel is pumped. Ultrasound probe shows a prostate during brachytherapy procedure with multiple needles inserted. Note, this patient has an asymmetrical urethra making the determination of its location under ultrasound more challenging. A. Contrast lumen without aerated gel. B. Contrast lumen with aerated gel. C. Video animation showing the prostate with aerated gel pumped continuously in and out of the contrast lumen, allowing easy identification of the urethra (available in the supplementary data, Appendix A). (Images generated by Ms. Terry Helms from the office of Design and Creative Services of MSKCC based on a description provided by the author, N.J.N.). © Memorial Sloan-Kettering Cancer Center, with permission.

Visualization of the prostatic urethra under ultrasound is important during invasive procedures such as prostate brachytherapy or biopsy. Injury to the prostatic urethra during these procedures is associated with increased toxicity [19]. During seed implantation for brachytherapy, delineation of the urethra is important as pretreatment planning constraints aim to keep UD5 (dose to 5% of the urethral volume) < 150% of the prescribed dose, and UD30 (dose to 30% of the urethral volume) < 125% [20], [21], [22], [23], [24]. For intraoperatively planned brachytherapy, a median increase of 30% in prostate volume due to edema has been reported immediately after needle insertion [25]. Delineation of the prostatic urethra after brachytherapy needles insertion is challenging (Fig. 3) because of difficulties in discriminating between intraprostatic calcifications, brachytherapy needles and the empty Foley catheter.

The Nasser – Zelefsky Ultrasound Catheter (NZUC) also has three lumens (Fig. 3), but differs from the NZCC, by the contrast used and the pump control. In the NZUC system, the first lumen fills a balloon with water to anchor the catheter to the bladder. The second lumen drains the bladder of urine. The third lumen has an external opening attached to an aerated gel pump and ends in a reservoir balloon that allows the pump to drive the aerated gel continuously in (Fig. 3B) and out of the lumen (Fig. 3A) while maintaining low internal pressure. This results in “flashing signal” on the US monitor screen at the location of the prostatic urethra, as a result of the continuous change in the density of the contrast lumen content between air and the gel, which has the same density as water (Fig. 3C). This facilitates continuous real-time urethral visualization under ultrasound.

## Discussion

Both Nasser-Zelefsky catheters can potentially improve precision of radiation delivery during EBRT and brachytherapy. Since the urethra traverses the prostate, once a catheter is inserted its location is constant relative to the prostate [26], [27]. The NZCC contains a pump that injects contrast into a catheter lumen in the prostatic urethra, allowing the computer system to “see” the urethra and use it to align the patient prior to each RT fraction so that the prostate is in the exact same distance from the isocenter for each RT treatment. Compared to fiducial markers, the number of registration points used to match the position of the prostate between the planning CT and the pretreatment images (CBCT) could be higher potentially increasing the image fusion accuracy, albeit with a potential drawback that the fusion points are all in relatively central locations within the prostate (since the urethra is usually a centrally-located structure).

Fiducial markers are usually inserted through transrectal route, a procedure associated with risk of infection [28]. Loh et al. reported that after fiducial marker insertion, 11.6% of the patients reported episodes of chills and fevers, 7.7% reported receiving antibiotics for urinary infection and 2.8% reported hospital admission for urosepsis related to the procedure [28]. Catheter insertion is less invasive than inserting fiducial markers. Moreover, fiducial markers can migrate, which can greatly reduce the accuracy of image guidance [29]. The NZCC also allows computerized couch control to fuse the automatically segmented urethra before treatment with the planning CT scan. This results in fewer manual manipulations and potential for human error while fine tuning the couch position before RT.

The accuracy of the fusion of the planning CT and the CBCT using the prostatic urethra to guide fusion, was reported to be high in previous studies [26], [27], although these studies used endorectal balloons in all patients. The ultimate validation study of the Nasser-Zelefsky Catheters would use fiducial markers and catheter to verify if matching the catheter in the prostatic urethra would also accurately match the fiducial markers. However, some of the prostate cancer patients naturally have calcifications in the prostate that are apparent on CT imaging and can be used as image-guidance markers in the absence of fiducial markers. We have initiated a study to test fusion accuracy in prostate cancer patients with prostatic calcifications treated with Foley catheter with IGRT using the prostatic urethra for image guidance.

As the CT slice thickness can be reduced to as thin as 1 mm [30], 10 points of reference could be generated for each 1 cm of prostatic urethra length. The location of the prostatic urethra is fixed compared to the prostate anatomy once a catheter is inserted [26], [27] and so issues of seed migration encountered with fiducial markers are eliminated with our technique. Moreover, visible artefacts on CT and CBCT scans are a known problem when using fiducial markers [31], and using a urethral catheter for image fusion rather than fiducial markers could potentially solve this problem. The NZCC could be incorporated into the Nasser-Zelefsky catheter for bladder filling [12] resulting in automatic localization of the prostate and consistent bladder filling using a single catheter.

Potential drawbacks of the proposed CT method for urethral localization include the double scanning used, which requires imaging both before and after contrast injection, doubling the radiation exposure to the patient from imaging studies. Pelvic CBCT is associated with a dose of 20–30 mGy per scan [32]. This could result, together with the CT simulation, in a radiation dose of 0.24–0.36 Gy from imaging for a course of 5 fractions SBRT, compared to 0.12–0.18 Gy in the current practice. Catheter placement prior to each visit also results in inconvenience to the patient and could be associated with urinary tract infection. Risk of infection may also be increased if the same catheter is left indwelling for multiple days without removal. These drawbacks will likely limit the use of this technology to prostate SBRT, in which radiation is provided in a limited number of fractions [4], [8], [11], [33], [34].

The injection of aerated gel into Foley catheter at time of US imaging for brachytherapy planning is a well-established technique [22], [23], [24], [35]. The NZUC improves on current methods by allowing continuous visualization of the urethra during the entire brachytherapy procedure, not only at time of image capturing. Also, the contrast is pumped automatically in and out of the catheter, eliminating the need for a surgical team member to inject the gel manually.

## Conclusions

The NZCC uses digital subtraction technology to detect discrete points in the prostatic urethra for localization of the prostate and to automatically position the patient accurately and reproducibly relative to the isocenter with less human labor. NZUC allows continuous visualization of the urethra under ultrasound, which may improve intra-procedure localization of the urethra to reduce urethral toxicity and potentially improve brachytherapy outcomes. These technologies should be validated and tested against current image-guidance techniques in clinical trials.
